# Behavioral Pathway between Social Support and Network, and Edentulism

**DOI:** 10.1177/00220345251329337

**Published:** 2025-05-04

**Authors:** F. Alobaidi, E. Heidari, W. Sabbah

**Affiliations:** 1 Faculty of Dentistry, Oral & Craniofacial Sciences, King’s College London, London, UK

**Keywords:** aging, dentition, health behavior, latent class analysis, structural equation modeling, social factors, socioeconomic factors

## Abstract

The aims of this study were to evaluate how behaviors cluster together and to investigate the relationship among cluster of behaviors, social support and network, socioeconomic factors, and edentulism in older English adults. Data on social factors (Wave 3, 2006/07), behaviors (Wave 5, 2010/11), and edentulism (Wave 7, 2014/15) were extracted from the English Longitudinal Study of Ageing (ELSA). Baseline demographic factors (gender, ethnicity, and age) were included. Latent class analysis (LCA) was conducted on 4 behaviors (smoking, alcohol intake, fruit and vegetable consumption, physical activity). Confirmatory factor analysis (CFA) was used to create 2 latent variables, social support and network (positive support, negative support, network), and socioeconomic factors (education, wealth, self-rated social status). Two models of structural equation modeling (SEM) were constructed to assess the direct and indirect effect of latent variables on edentulism. A total of 3,087 participants were included. In the LCA, a 2-class model was chosen: class 1 (risky) and class 2 (healthy). The first SEM model showed that social support and network was not linked directly to edentulism, but higher levels of social support and network predicted being dentate indirectly through cluster of behaviors. The second model additionally accounting for socioeconomic position showed that social support and network was not associated with edentulism directly or indirectly, but higher socioeconomic position predicted directly and indirectly being dentate. In both models, cluster of behaviors was associated with edentulism. The result of this study clearly shows that cluster of behaviors mediate the relationship between each of social support and socioeconomic position and edentulism. Actions to improve socioeconomic conditions might have strong effects on changing behaviors and improved oral health.

## Introduction

As the population ages, the prevalence of tooth loss has increased, with rates peaking among adults 60 y and older around the world (Kassebaum et al. 2014; Bernabe et al. 2020). The loss of natural permanent teeth (edentulism) can greatly affect the quality of life of older adults, influencing their lifestyle, social life, and dietary options (Public Health England 2015).

An important determinant of oral health is social support and network. Social networks describe the connections that individuals have with their community members (Berkman and Syme 1979), while social support refers to the emotional and physical support provided by these connections (Lin et al. 1979; Cooke et al. 1988). The interaction between these social relationships can result in numerous health outcomes (Hunter et al. 2013). Social networks and social support have been identified as risks for oral diseases and tooth loss (Aida et al. 2016; Vettore et al. 2016; Laniado et al. 2023). Moreover, individuals with lower socioeconomic status (SES) have poorer oral health compared with those from more advantaged socioeconomic backgrounds (Vettore et al. 2016).

Social factors can influence oral health outcomes through different mediators, including psychosocial, behavioral, and various clinical factors (such as chronic conditions) (Watt and Sheiham 2012). The behavioral hypothesis highlights the increased probability of people engaging in unhealthy behaviors, especially individuals coming from low socioeconomic backgrounds (Sisson 2007). However, these behaviors often co-occur in groups and clusters (Meader et al. 2016; Austregésilo et al. 2019). A systematic review found evidence of clustering of different risky behaviors (such as smoking, alcohol consumption, and poor diet), with socioeconomic conditions being the biggest predictor of engaging in multiple risky behaviors (Meader et al. 2016). This indicates that engaging in multiple risky behaviors is more common among lower socioeconomic groups, potentially affecting general and oral health. The behavioral pathway could explain the socioeconomic inequalities in oral health, as health-risk behaviors typically cluster among those from low socioeconomic backgrounds (Sanders et al. 2005).

Edentulism can be influenced by multiple behaviors, even if these behaviors are not directly linked to tooth loss. These associations are rarely addressed in the literature, particularly using longitudinal data. A systematic review showed that previous studies have focused on exploring the association of multiple behaviors with tooth loss rather than examining clusters of behaviors (Alobaidi et al. 2024). There is a lack of studies that investigated the role of clusters of behaviors in the relationship between socioeconomic factors, social network/support, and edentulism. Therefore, the aims of this study were (1) to assess how behaviors cluster together among older adults, (2) to explore the direct relationship between social factors and edentulism, and (3) to assess the indirect effect of social factors on edentulism through clusters of behaviors.

## Methodology

### Study Population

Data from the English Longitudinal Study of Ageing (ELSA) were used from Wave 3 (2006/07), Wave 5 (2010/11), and Wave 7 (2014/15). ELSA is a comprehensive cohort study started in 2002 and included more than 12,000 community-dwelling English adults aged 50 y and older (Banks et al. 2021). The initial ELSA sample was drawn from the Health Survey of England conducted in 1998, 1999, and 2001. The survey uses multistage stratified sampling, dividing the population into strata and randomly selecting participants using computer-generated techniques for comprehensive representation. Ethical approval for ELSA waves was granted by the Multicentre Research and Ethics Committee (MREC/01/2/91).

Participants were interviewed using computer-assisted personal interview, followed by self-completion questionnaire. The survey collected data on health-related behaviors, including smoking, alcohol intake, fruit and vegetable consumption, and physical activity as well as data on social support, social networking, and socioeconomic conditions. Wave 3 was chosen as the baseline for this study, as it was the first wave that included measurements of complete tooth loss. Wave 7 was chosen as the follow-up for the study as it was the last wave that included tooth loss.

### Variables

Edentulism was self-reported based on the presence or absence of natural teeth. The variable was included from Wave 3 (baseline) and Wave 7 (follow-up). The variable was categorized as edentate versus dentate.

Four health-related behaviors were included from Wave 5 (smoking, alcohol intake, fruit and vegetable consumption, and physical activity). All behaviors were used as dichotomous variables, smoking (smokers, nonsmokers), alcohol intake (14 units or less per week, more than 14 units) (Department of Health and Social Care 2016), fruit and vegetable (less than 5 portions per day, 5 portions or more) (Kojima et al. 2020), and physical activity (no or low activity per week, moderate or high activity).

Questions on social factors from Wave 3 covered relationships with spouse/partner, children, friends, and extended family members. Positive social support was addressed through 3 questions (how much they understand, rely on, and can open up to). Responses were recorded on a 4-item scale and summed to create a total score ranging from 0 to 36. Negative social support was addressed through 3 questions (how much they criticized, let you down, and made you feel nervous). Responses were scored as described and were summed and reversed into a scale ranging from 0 to 28 (Liao and Scholes 2017). Social network was measured by asking participants how many close relationships they had with their children, family, and friends. Emotional closeness with a spouse was also assessed (how close is your relationship to your spouse) on a scale ranging from 0 (*not at all*) to 3 (*a lot*), with all responses combined into a final score ranging from 0 to 28 (Rouxel et al. 2015).

The socioeconomic factor from Wave 3 was included. Participants’ education, wealth, and self-rated social status were included. Education was categorized as less than O-level or equivalent, O-level or equivalent, and higher than A-level. O-level is equivalent to a high school diploma, whereas A-level is equivalent to advanced high school diplomas or preuniversity qualifications (Government of the United Kingdom [GOV UK] 2021). Wealth was measured on a scale ranging from 0 to 5, with higher values indicating greater wealth. The total wealth calculation included the combined value of housing, financial assets, and physical possessions owned by the household, with debts excluded (Demakakos et al. 2016). Self-rated social status was measured on a scale from 5 to 100, with a higher value indicating higher social status (Coustaury et al. 2023). Baseline demographics such as gender and age (in Wave 3 and Wave 5) were included.

### Statistical Analysis

Latent class analysis (LCA) was conducted to identify clustering patterns of health-related behaviors among older adults. LCA provides a model-based method, using multiple observable categorical factors to identify underlying subgroups within a population. The goodness-of-fit indices, which gives the adjusted values for the log likelihood such as the Akaike information criterion, Bayesian information criterion, and adjusted Bayesian information criterion, were used to determine the optimal number of clusters for the data (Schwarz 1978; Akaike 1987). Lower values were considered to represent the best model fit for the data. Confirmatory factor analysis (CFA) was conducted to assess the measurement model for the 2 latent variables: social support and network (positive/negative social support and social network) and socioeconomic factors (wealth, education, and self-rated social status). Structural equation modeling (SEM) was used to investigate the direct association between social support and network, socioeconomic factors, and edentulism as well as the indirect impact through cluster of behaviors. The model was adjusted for demographic variables and edentulism at baseline. The maximum likelihood with missing values estimation method was used in CFA and SEM analysis. The Tucker-Lewis Index (TLI), the Comparative Fit Index (CFI), and the root mean square error of approximation (RMSEA) were used to assess the goodness of fit. A satisfactory model fit is indicated by an RMSEA value <0.05 and a CFI and TLI >0.90 (Kline 2011). A sensitivity analysis using smoking instead of cluster of behaviors was conducted to address the concern regarding behaviors not directly linked to edentulism. Another sensitivity analysis was conducted excluding edentate participants at baseline. Standardized coefficients (SC), 95% confidence intervals (CIs), and *P* values were reported. All analyses were conducted using Mplus version 8.10 software.

### Results

After exclusion of participants with missing data and those lost during the follow-up period (died or moved) (*n* = 3,863), a total of 3,087 participants were included in the analysis (Table 1). A flow chart is presented in Appendix Figure 1. At baseline, 54% were females, with an age average of 63 y. Most participants had moderate wealth, and almost all had some natural teeth (92%). Forty percent of participants had an education higher than A-level, whereas 60% had O-level education or less. Most participants had a higher score in positive and negative social support and lower score in social network. There was a significant difference between the included and excluded samples; therefore, this analysis may have underestimated the longitudinal association between social factors and edentulism as the analytical sample represents individuals with more favorable sociodemographic and oral health characteristics.

**Table 1.** Descriptive Statistics of the Sample (*n* = 3,087).

Variable	Baseline Sample (*N* = 6,950)	Excluded Sample (*n* = 3,863)	Included Sample (*n* = 3,087)	
	Percentage/Mean	Percentage/Mean	Percentage/Mean	*P* Value^a^
Gender				
Male	45%	44%	46%	
Female	55%	56%	54%	<0.059
Ethnicity				
White	98%	98%	99%	
Non-White	2%	2%	1%	<0.001
Education				
Less than O-level or equivalent	41%	50%	30%	
O-level or equivalent	26%	23%	30%	
Higher than A-level	33%	27%	40%	<0.001
Age (at Wave 3)	62.6	67.9	62.6	<0.001
Wealth (at Wave 3)	3.5	2.9	3.5	<0.001
Self-rated social status (Wave 3)	61.5	57.0	61.5	<0.001
Positive social support (Wave 3)	24.1	22.3	24.1	<0.001
Negative social support (Wave 3)	20.9	6.5	20.9	<0.005
Social network (Wave 3)	9.5	8.8	9.5	<0.001
Edentulism (Wave 3)				
Yes	84%	21%	8%	
No	15%	79%	92%	<0.001
Fruits and vegetable (Wave 5)				
Less than 5 portions per day	31%	24%	40%	
5 portion or more per day	42%	27%	60%	<0.001
Missing	27%	49%		
Smoking (Wave 5)				
Not smokers	70%	54%	90%	
Smokers	9%	8%	10%	<0.001
Missing	21%	38%		
Physical activity (Wave 5)				
No or low activity per week	20%	23%	15%	
Moderate or high activity per week	60%	40%	85%	<0.001
Missing	20%	37%		
Alcohol intake (Wave 5)				
14 or fewer units per week	56%	30%	87%	
More than 14 units per week	7%	3%	13%	<0.001
Missing	37%	67%		
Cluster of behaviors (Wave 5)				
Class 1 (Risky)			7%	
Class 2 (Healthy)			93%	
Age at (Wave 5)	66.7	71.9	66.7	
Missing	40%	40%		
Edentulism (Wave 7)				
Yes	4%	1%	9%	
No	44%	6%	91%	<0.05
Missing	52%	93%		

a *P* value from the chi-square test and *t* test.

One to 4 classes were generated using LCA based on 4 health-related behaviors. According to the goodness-of-fit indices, the 2-class model was the best fit for the data (Appendix Table 1). Most of the participants (93%) were in class 2 (healthy), while only 7% were in class 1 (risky). Table 2 shows the estimated probabilities of health-related behaviors specific to each cluster. Participants in the risky cluster had a low probability of consuming more than 5 portions of fruit and vegetables, a moderate probability of being a smoker, a high probability of engaging in high physical activity, and a moderate probability of drinking more than 14 units per week. In contrast, participants in the healthy cluster had the highest probability of consuming more than 5 portions of fruit and vegetables, the lowest probability of smoking, the highest probability of engaging in high physical activity, and a low probability of drinking more than 14 units per week. Most participants in the risky cluster were male with less than an O-level education, while most of the participants in the healthy cluster were female with a higher level of education. Most participants in both clusters were White and had some natural teeth. The mean age was 59 y in the risky cluster and 62 y in the healthy cluster. The mean scores for social support (both positive and negative) and self-rated social status were relatively high in both clusters, while social network scores were low across all classes.

**Table 2.** Latent Class Probabilities and Class Descriptives (*n* = 3,087).

Two-Class Model	Class 1 (Risky)	Class 2 (Healthy)
Item-response probabilities		
Fruits and vegetables		
Fewer than 5 portions per day	80%	31%
5 portion or more per day	20%	69%
Smoking		
Not smokers	69%	96%
Smokers	31%	4%
Physical activity		
No or low activity per week	27%	13%
Moderate or high activity per week	73%	87%
Alcohol intake		
14 or fewer units per week	78%	90%
More than 14 units per week	22%	10%
Class descriptive percentages		
Gender		
Male	56%	46%
Female	44%	54%
Education		
Less than O-level or equivalent education	40%	29%
O-level or equivalent education	35%	29%
Higher than A-level education	25%	42%
Edentulism		
Yes	11%	8%
No	89%	92%
Ethnicity		
White	99%	99%
Non-White	1%	1%
Class descriptive mean		
Age	59.5	62.8
Wealth	2.7	3.5
Self-rated social status	56.4	61.8
Positive social support	22.3	24.2
Negative social support	20.2	20.9
Social network	8.8	9.5

CFA was conducted to create the latent variables. The SCs for each domain are tabulated in Appendix Table 2. Most item loadings were significant (<0.001), and the latent factors were highly correlated. Model 1 includes only social support and network variables (social support positive, social support negative, social network), while model 2 includes 2 latent variables, social support and network, and socioeconomic factors (education, wealth, self-rated social status).

Table 3 shows the SEM model with 1 latent variable (social support and network) and provides the SCs, including the direct, indirect, and total effects between social support and network, cluster of behaviors, and edentulism (Appendix Fig. 2). The model presented good fit values and were adjusted for demographics at baseline, edentulism at baseline, and age at Wave 5. Social support and network at Wave 3 were not linked to edentulism at Wave 7. However, greater social support and network indirectly predicted being dentate at Wave 7 (SC = 0.001). In addition, being in a healthier cluster of behaviors at Wave 5 directly predicted being dentate at Wave 7 (SC = 0.017). Higher levels of social support and network at Wave 3 also directly predicted being in the healthier cluster of behaviors at Wave 5 (SC = 0.075).

**Table 3.** Structural Equation Modeling Pathway for the Association between Social Support and Network and Edentulism (Wave 3 to Wave 7) (*n* = 3,087).

Variable	SC	95% CI	*P* Value
Direct effect to cluster of behaviors			
Social support and network	0.075	(0.032, 0.118)	<0.001
Direct effect to edentulism			
Social support and network	0.000	(−0.011, 0.021)	0.934
Cluster of behaviors	0.017	(0.008, 0.026)	<0.001
Edentulism Wave 3	0.098	(0.090, 0.105)	<0.001
Gender	−0.006	(−0.017, 0.005)	0.272
Age at Wave 3	−0.086	(−0.078, 0.251)	0.303
Ethnicity	0.987	(0.983, 0.990)	<0.001
Age at Wave 5	−0.103	(−0.268, 0.062)	0.223
Indirect effect to edentulism (through cluster of behaviors)			
Social support and network	0.001	(0.000, 0.002)	<0.05
Total effect to edentulism (direct + indirect)			
Social support and network	0.002	(−0.010, 0.0013)	0.765
Model fit			
RMSEA	0.021	(0.014, 0.028)	
CFI	1.00		
TLI	1.00		

CFI, comparative fit index; CI, confidence interval; RMSEA, root mean square error of approximation; SC, standardized coefficient; TLI, Tucker–Lewis index.

Table 4 presents the SEM model including 2 latent variables (social support and network and socioeconomic factor). The Figure illustrates the significant pathways in the final structural model. In this model, social support and network at Wave 3 were not linked directly or indirectly to edentulism at Wave 7. However, greater SES at Wave 3 directly predicted being in a healthier cluster of behaviors at Wave 5 (SC = 0.176) and being dentate at Wave 7 (SC = 0.020). Higher levels of socioeconomic factors at Wave 3 also indirectly predicted being dentate at Wave 7 (SC = 0.002). Finally, being in a healthier cluster of behaviors at Wave 5 directly predicted being dentate at Wave 7 (SC = 0.014). When we used smoking instead of cluster of behaviors in a sensitivity analysis, the results were similar to those observed in the original analysis (Appendix Table 3). Similarly, the results from the sensitivity analysis where we excluded edentate participants at baseline were similar to the results in the original model (Appendix Table 4). The analysis was conducted without accounting for the stratified and clustered design; however, a sensitivity analysis was performed and yielded similar results (Appendix Table 5).

**Table 4.** Structural Equation Modeling Pathway for the Association between Social Factors, Socioeconomic Factors, and Edentulism (Wave 3 to Wave 7) (*n* = 3,087).

Variable	SC	95% CI	*P* Value
Direct effect to cluster of behaviors			
Social support and network	0.029	(−0.014, 0.073)	0.185
Socioeconomic factors	0.176	(0.134, 0.219)	<0.001
Direct effect to social support and network			
Socioeconomic factors	0.259	(0.208, 0.310)	<0.001
Direct effect to edentulism			
Social support and network	−0.005	(−0.017, 0.008)	0.472
Socioeconomic factors	0.020	(0.006, 0.034)	<0.005
Cluster of behaviors	0.014	(0.005, 0.021)	<0.005
Edentulism Wave 3	0.098	(0.090, 0.105)	<0.001
Gender	−0.006	(−0.017, 0.005)	0.272
Age at Wave 3	0.086	(−0.078, 0.251)	0.303
Ethnicity	0.987	(0.983, 0.990)	<0.001
Age at Wave 5	−0.103	(−0.268, 0.062)	0.223
Indirect effect to edentulism (through cluster of behaviors)			
Social support and network	0.000	(0.000, 0.001)	0.236
Socioeconomic factors	0.002	(0.001, 0.004)	<0.005
Total effect to edentulism (direct + indirect)			
Social support and network	−0.004	(−0.017, 0.008)	0.514
Socioeconomic factors	0.022	(0.009, 0.036)	<0.005
Model fit			
RMSEA	0.043	(0.038, 0.047)	
CFI	0.99		
TLI	0.99		

CFI, comparative fit index; RMSEA, root mean square error of approximation; SC, standardized coefficient; TLI, Tucker–Lewis index.

**Figure.**
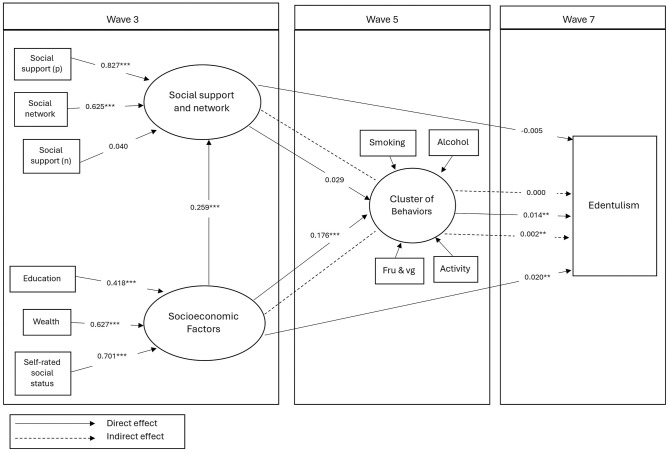
The significant pathway of the final structural model between social support/network and socioeconomic variables and edentulism over time.

## Discussion

This study explored how behaviors cluster among older English adults and examined the behavioral pathway between social support and network, socioeconomic factors and edentulism. Good social support and network were indirectly important determinants of being dentate after 9 y. After accounting for socioeconomic factors, the relationship between social support/network and edentulism was attenuated and lost statistical significance, highlighting the role of higher socioeconomic position. In addition, being in a healthy behavior cluster was directly linked to being dentate.

The hypothesis that social support and network affect edentulism through clusters of behaviors was partially confirmed. While social support and network was indirectly linked to edentulism, the direct effect was not significant. A previous study using an SEM model showed that poor social ties (social support and network) and lower social position were directly and indirectly (through smoking and psychological distress) associated with tooth loss (Vettore et al. 2016). In another study, social network was significantly associated with a higher number of remaining teeth among Japanese adults (Aida et al. 2016). The study also adjusted for oral health behaviors (dental visits, interdental devices, fluoride toothpaste use), showing the attenuation of the association after these adjustments. Another study found that lower social support was related to more tooth loss and dental caries among American adults (Laniado et al. 2023).

As previous studies showed risk behaviors commonly coexist and have a significant impact on health (Meader et al. 2016). In relation to oral health, the clustering of different behaviors (such as poor diet, irregular brushing, smoking, and drinking) can increase the risk of oral health issues (Austregésilo et al. 2019). In the current analysis, clusters of behaviors were directly associated with edentulism, and individuals in the healthy cluster were more likely to retain their natural teeth. A recent systematic review indicated that engaging in multiple risky behaviors (oral and general behaviors) is associated with having fewer teeth (Alobaidi et al. 2024). Habits such as poor oral hygiene, smoking, and lower consumption of fruits and vegetables can increase the chances of periodontal disease and caries and lead to tooth loss (Burt et al. 1990; Kressin et al. 2003; Skoczek-Rubińska et al. 2018). One study showed a decreased risk of tooth loss among individuals who engaged in multiple favorable oral health behaviors (toothbrushing, flossing, and regular dental prophylaxis) (Kressin et al. 2003). This emphasizes that the clustering of behaviors can have an impact on edentulism. Studies confirmed that individuals from lower SES tend to cluster together and commonly engage in multiple risky behaviors (Sanders et al. 2005). Given that participants with low SES frequently experience more obstacles to oral health, they might benefit more from social support. Exclusion of those participants in this analysis may have limited the study’s ability to observe stronger effects of social support on edentulism. Selection bias may have been induced by this exclusion, understating the contribution of social support to reducing inequities in oral health among underprivileged groups.

The effect of socioeconomic factors was evident in the current study, showing the strong association of these factors with edentulism directly and indirectly through behaviors. SES can influence access to faculties (Bas and Azogui-Lévy 2022). Urban areas and wealthier communities have better access to comprehensive dental services compared with rural or lower-income areas (Khan et al. 2017). In addition, higher socioeconomic levels enable individuals to choose advanced dental treatment, which can preserve natural teeth (Correa et al. 2013). The indirect effect of socioeconomic factors could be highlighted in its influence on knowledge and behaviors (Çirakoğlu and Gökcek 2021), which often correlate with better oral hygiene practices that reduce the risk of tooth loss (Giusti et al. 2014). Another study found that higher levels of socioeconomic factors were associated directly and indirectly with tooth loss through healthy diet (Stein et al. 2021). A different study showed that early and late life SES were linked to tooth loss (Celeste et al. 2020). This highlights the importance of exploring the life-course approach, which was not done in this study. Future studies should explore this perspective to better understand the cumulative effects of social inequalities on oral health.

Although this study examined the behavioral pathway between social factors and edentulism, social support can affect health in other ways. One potential pathway is biological, in which social support affects the physiological process linked to stress and immune responses (Uchino et al. 2018). Another pathway is the psychosocial pathway, which includes the cognitive and emotional states of individuals such as self-esteem and social skills (Uchino et al. 2012). Adequate social support and networks can provide individuals with different resources such as financial and physical assistance for accessing dental care (Amininia et al. 2023) and encourage positive behaviors.

There are some limitations of the study worth noting. First, reliance on self-reported data may introduce recall bias, although such measures are widely used in epidemiological research. Second, the analyses were based initially on a representative sample with high response rates; however, the exclusion of a large number of participants due to missing data, particularly those from disadvantaged backgrounds, may have introduced some bias and affected the generalizability of the findings. Third, the survey lacks key behaviors such as dental visits and oral hygiene, which strongly correlate with oral health. Fourth, detailed measures of tooth loss were unavailable due to the limited dental data in ELSA. Fifth, ethnic disparities could not be analyzed as these data were excluded for privacy reasons. Finally, the study used a complete case (CC) analysis and excluded cases that differed from included cases in terms of SES and health behaviors, suggesting that the CC approach may have underestimated the effect of these variables on the outcome.

The findings suggest that dental health initiatives should focus on distal factors to reduce social inequalities in oral health. The study uniquely included health behaviors that are not directly linked to oral health, such as physical activity. Using clustering of behaviors rather than examining each behavior separately was a strength, as it allowed us to see how behaviors group together and the effect of these clusters. Policy makers should implement strategies that address multiple behaviors and provide resources to support healthier choices among the aging population. Moreover, understanding the factors behind these behaviors is key to effective change, and addressing social inequalities is vital for successful health interventions.

In conclusion, this study demonstrated that behaviors tend to cluster within the same population, and these clusters are associated with social factors and edentulism. Therefore, the indirect association through the behavioral pathway was confirmed. To effectively reduce oral health inequalities, dental health initiatives must address multiple behaviors and the underlying social factors for the aging population.

## Authors’ Contributions

F. Alobaidi, contributed to the development and structuring of the research framework, conducted the data analysis and interpreting the results, drafted the initial version of the manuscript, outlined the key findings and interpretations of the research; E. Heidari, contributed to the interpretation of the data, provided valuable insights to enhance the understanding of the findings, revised and edited the manuscript, offered valuable insights to enhance the interpretation and presentation of the results; W. Sabbah, developed the research design, offered critical insights to refine the study’s methodology and objectives, contributed to the data analysis, provided critical insights to ensure accurate interpretation of the findings, contributed to the revision and editing of the manuscript, provided critical feedback to refine the analysis and improve the clarity of the findings. All authors gave final approval and agree to be accountable for all aspects of the work.

## Supplemental Material

sj-docx-1-jdr-10.1177_00220345251329337 – Supplemental material for Behavioral Pathway between Social Support and Network, and Edentulism Supplemental material, sj-docx-1-jdr-10.1177_00220345251329337 for Behavioral Pathway between Social Support and Network, and Edentulism by F. Alobaidi, E. Heidari and W. Sabbah in Journal of Dental Research
