# Supplementary material for: Behavioral Pathway between Social Support and Network, and Edentulism
Source: J Dent Res. 2025 May 4;104(10):1077–84. doi: 10.1177/00220345251329337 (PMC12301509; doi:10.1177/00220345251329337)
Supplement: sj-docx-1-jdr-10.1177_00220345251329337 – Supplemental material for Behavioral Pathway between Social Support and Network, and Edentulism [file sj-docx-1-jdr-10.1177_00220345251329337.docx]

**Behavioral Pathway Between Social Support and Network, and Edentulism.**

Fatimah Alobaidi1, Ellie Heidari1, Wael Sabbah1

**Appendix Methods: Statistical analyses**

**Appendix Figure 1 -** Flow chart of the analytical sample (Wave 3 - Wave 7).

**
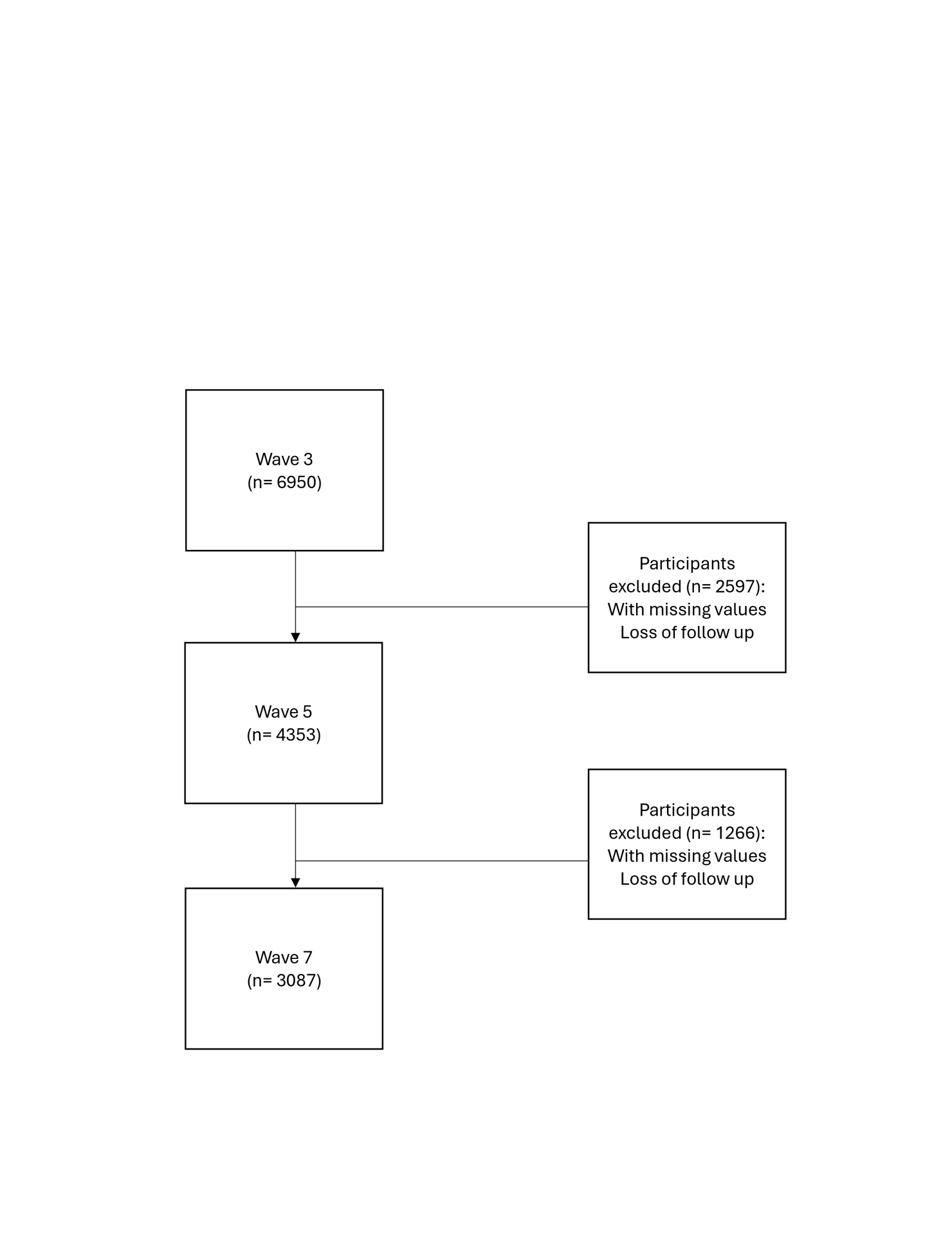
**

**Appendix Table 1** - Model fit indices for the latent class analysis (n = 3087).

| Classes | FP | AIC | BIC | A-BIC | Entropy | LMR p-value | BLRT p-value |
| --- | --- | --- | --- | --- | --- | --- | --- |
| Class 1 | 4 | 11086.219 | 11110.359 | 11097.649 | - | - | - |
| Class 2 | 9 | 11004.073 | 11058.388 | 11029.791 | 0.488 | 0.00 | 0.00 |
| Class 3 | 14 | 11007.977 | 11092.466 | 11047.983 | 0.762 | 0.04 | 0.30 |
| Class 4 | 19 | 11015.364 | 11130.028 | 11069.658 | 0.724 | 0.61 | 0.33 |

FP, free parameter; AIC, Akaike Information Criterion; BIC, Bayesian Information Criterion; A-BIC, Adjusted Bayesian Information Criterion; LMR, Likelihood Ratio Test; BLRT, Boosted Likelihood Ratio Test.

**Appendix Table 2 -** Measurement model (Confirmatory Factor Analysis) standardized estimates for latent variables, 95% confidence interval and p-value for model 1 and model 2 (n = 3087).

| Latent variable | | Model 1 | | | Model 2 | | |
| --- | --- | --- | --- | --- | --- | --- | --- |
| Social support and network | | SC | 95% CI | P-value | SC | 95% CI | P-value |
|  | Social support (positive) | 0.809 | (0.558, 1.059) | <0.001*** | 0.827 | (0.733, 0.921) | <0.001 |
|  | Social support (negative) | 0.027 | (-0.013, 0.067) | 0.179 | 0.040 | (0.000, 0.080) | 0.052 |
|  | Social network | 0.642 | (0.442, 0.842) | <0.001*** | 0.625 | (0.553, 0.0.698) | <0.001 |
| Socioeconomic factors | |  |  |  |  |  |  |
|  | Education |  |  |  | 0.418 | (0.376, 0.461) | <0.001 |
|  | Wealth |  |  |  | 0.627 | (0.582, 0.672) | <0.001 |
|  | Self-rated social status |  |  |  | 0.701 | (0.656, 0.746) | <0.001 |
| Model fit indices | |  |  |  |  |  |  |
|  | RMSEA | 0.021 | (0.014, 0.028) |  | 0.043 | (0.038, 0.047) |  |
|  | CFI | 1.00 |  |  | 0.99 |  |  |
|  | TLI | 1.00 |  |  | 0.99 |  |  |

**Appendix Figure 2 -** Significant pathway of the structural model between social support/ network and edentulism over time.

**
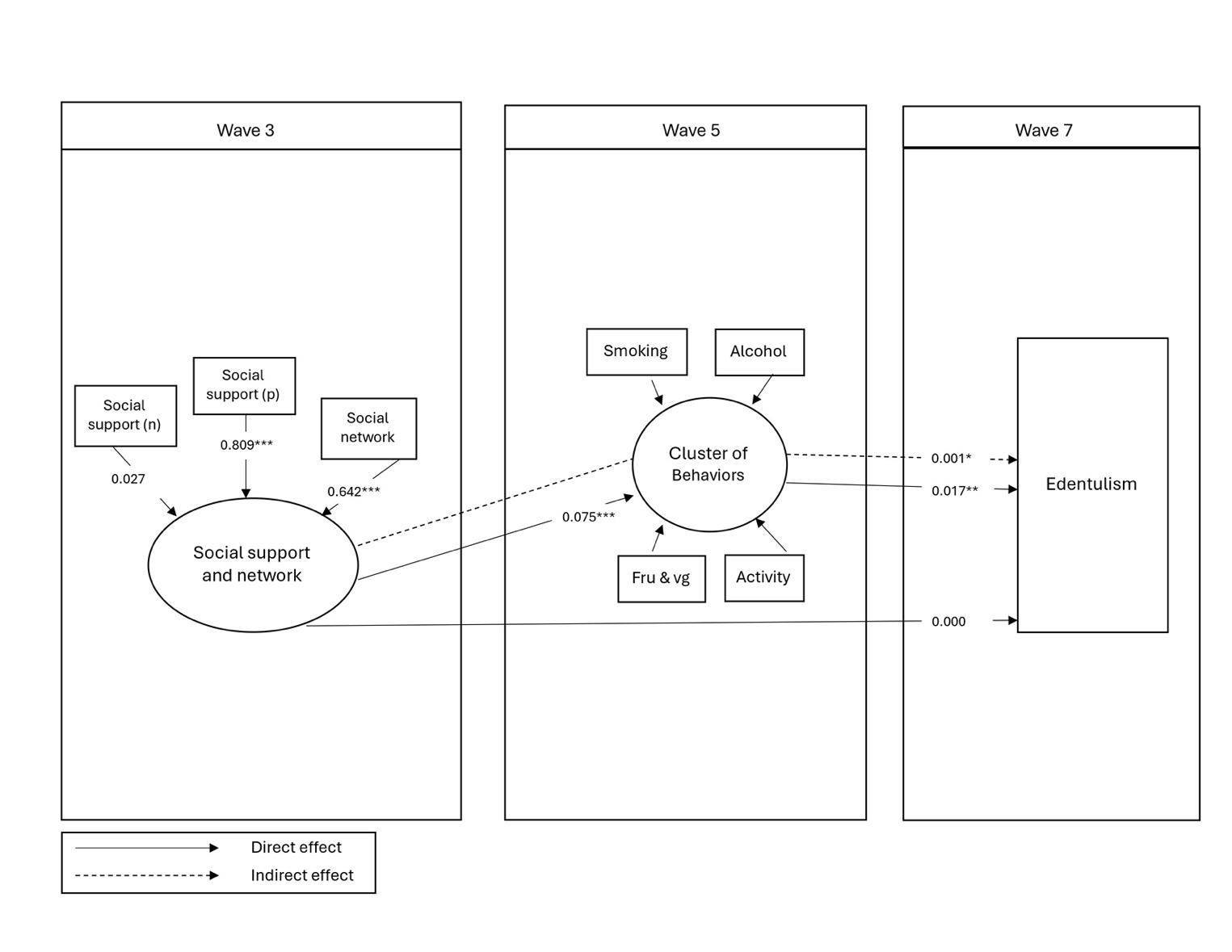
**

**Appendix Table 3 -** SEM pathway for the association between social factors, socioeconomic factors and edentulism through smoking (wave 3 to wave 7) (n = 3087).

| Variables | | Coefficient | 95%CI | P-value |
| --- | --- | --- | --- | --- |
| Direct effect to Smoking | |  |  |  |
|  | Social support and network | -0.014 | (-0.056, 0.028) | 0.508 |
|  | Socioeconomic factors | -0.194 | (-0.238, -0.149) | <0.001 |
| Direct effect to Social support and network | |  |  |  |
|  | Socioeconomic factors | 0.260 | (0.208, 0.310) | <0.001 |
| Direct effect to Edentulism | |  |  |  |
|  | Social support and network | -0.004 | (-0.017, 0.008) | 0.494 |
|  | Socioeconomic factors | 0.020 | (0.006, 0.034) | <0.01 |
|  | Smoking | -0.013 | (-0.021, -0.004) | <0.01 |
|  | Edentulism Wave 3 | 0.098 | (0.090, 0.105) | <0.001 |
|  | Gender | -0.006 | (-0.017, 0.005) | 0.272 |
|  | Age at Wave 3 | 0.086 | (-0.078, 0.251) | 0.303 |
|  | Ethnicity | 0.987 | (0.983, 0.990) | <0.001 |
|  | Age at Wave 5 | -0.103 | (-0.268, 0.062) | 0.223 |
| Indirect effect to Edentulism (through smoking) | |  |  |  |
|  | Social support and network | 0.000 | (0.000, 0.001) | 0.523 |
|  | Socioeconomic factors | 0.002 | (0.001, 0.004) | <0.01 |
| Total effect to Edentulism (direct + indirect) | |  |  |  |
|  | Social support and network | -0.004 | (-0.017, 0.008) | 0.512 |
|  | Socioeconomic factors | 0.022 | (0.009, 0.036) | <0.01 |
| Model fit | |  |  |  |
|  | RMSEA | 0.042 | (0.038, 0.047) |  |
|  | CFI | 0.99 |  |  |
|  | TLI | 0.99 |  |  |

RMSEA, root mean square error of approximation; CFI, comparative fit index; TLI, Tucker–Lewis index.

**Appendix Table 4 –** Class distribution of LCA and the SEM pathway for the association between social factors, socioeconomic factors and edentulism through cluster of behaviors after excluding edentate participants at baseline (wave 3 to wave 7) (n = 2830).

| Class Item-response Probabilities | | | Class 1 (Healthy) | Class 2 (Risky) |
| --- | --- | --- | --- | --- |
| Fruits and vegetable | | < 5 | 30% | 81% |
|  | | ≥ 5 | 70% | 18% |
| Smoking | | No | 96% | 68% |
|  | | Yes | 4% | 32% |
| Physical activity | | No or low | 12% | 25% |
|  | | Moderate or high | 88% | 75% |
| Alcohol intake | | ≤ 14 | 89% | 76% |
|  | | > 14 | 11% | 24% |
|  | | | | |
| Variables | | Coefficient | 95%CI | P-value |
| Direct effect to Cluster of behaviors | |  |  |  |
|  | Social support and network | -0.017 | (-0.062, 0.029) | 0.468 |
|  | Socioeconomic factors | -0.190 | (-0.234, -0.145) | <0.001 |
| Direct effect to Social support and network | |  |  |  |
|  | Socioeconomic factors | 0.260 | (0.208, 0.310) | <0.001 |
| Direct effect to Edentulism | |  |  |  |
|  | Social support and network | -0.002 | (-0.031, 0.028) | 0.917 |
|  | Socioeconomic factors | 0.041 | (0.008, 0.075) | <0.05 |
|  | Cluster of behaviors | -0.022 | (-0.041, -0.004) | <0.05 |
|  | Gender | -0.004 | (-0.031, 0.023) | 0.747 |
|  | Age at Wave 3 | 0.326 | (-0.050, 0.703) | 0.089 |
|  | Ethnicity | 0.974 | (0.971, 0.976) | <0.001 |
|  | Age at Wave 5 | -0.349 | (-0.727, 0.029) | 0.070 |
| Indirect effect to Edentulism (through cluster) | |  |  |  |
|  | Social support and network | 0.000 | (-0.001, 0.001) | 0.495 |
|  | Socioeconomic factors | 0.004 | (0.001, 0.008) | <0.05 |
| Total effect to Edentulism (direct + indirect) | |  |  |  |
|  | Social support and network | -0.001 | (-0.031, 0.029) | 0.937 |
|  | Socioeconomic factors | 0.045 | (0.013, 0.078) | <0.01 |
| Model fit | |  |  |  |
|  | RMSEA | 0.035 | (0.031, 0.041) |  |
|  | CFI | 0.99 |  |  |
|  | TLI | 0.99 |  |  |

RMSEA, root mean square error of approximation; CFI, comparative fit index; TLI, Tucker–Lewis index.

**Appendix Table 5** - SEM pathway for the association between social factors, socioeconomic factors and edentulism (wave 3 to wave 7) after accounting for clustering and stratification (n = 3087).

| Variables | | Coefficient | 95%CI | P-value |
| --- | --- | --- | --- | --- |
| Direct effect to Cluster of behaviors | |  |  |  |
|  | Social support and network | 0.030 | (-0.014, 0.073) | 0.184 |
|  | Socioeconomic factors | 0.176 | (0.132, 0.220) | <0.001 |
| Direct effect to Social support and network | |  |  |  |
|  | Socioeconomic factors | 0.258 | (0.206, 0.329) | <0.001 |
| Direct effect to Edentulism | |  |  |  |
|  | Social support and network | -0.004 | (-0.017, 0.008) | 0.184 |
|  | Socioeconomic factors | 0.020 | (0.006, 0.034) | <0.05 |
|  | Cluster of behaviors | 0.041 | (0.005, 0.023) | <0.05 |
|  | Edentulism Wave 3 | 0.098 | (0.090, 0.106) | <0.001 |
|  | Gender | -0.006 | (-0.017, 0.005) | 0.264 |
|  | Age at Wave 3 | 0.086 | (-0.095, 0.268) | 0.451 |
|  | Ethnicity | 0.987 | (0.983, 0.702) | <0.001 |
|  | Age at Wave 5 | -0.103 | (-0.285, 0.080) | 0.271 |
| Indirect effect to Edentulism (through cluster of behaviors) | |  |  |  |
|  | Social support and network | 0.000 | (0.000, 0.001) | 0.235 |
|  | Socioeconomic factors | 0.022 | (0.008, 0.036) | <0.005 |
| Total effect to Edentulism (direct + indirect) | |  |  |  |
|  | Social support and network | -0.004 | (-0.017, 0.009) | 0.535 |
|  | Socioeconomic factors | 0.002 | (0.001, 0.004) | <0.005 |
| Model fit | |  |  |  |
|  | RMSEA | 0.043 | (0.038, 0.047) |  |
|  | CFI | 0.99 |  |  |
|  | TLI | 0.99 |  |  |
